# Osteopathic Manipulative Treatment for Complex Regional Pain Syndrome: A Scoping Review of Current Evidence

**DOI:** 10.7759/cureus.106748

**Published:** 2026-04-09

**Authors:** Alexander Ponce, Peter Nguyen, Jonathan Reves

**Affiliations:** 1 General Surgery, Central Surgical Associates, Jackson, USA; 2 Internal Medicine, Mississippi Baptist Medical Center, Jackson, USA; 3 Internal Medicine, William Carey University College of Osteopathic Medicine, Hattiesburg, USA; 4 Physical Medicine and Rehabilitation, William Carey University College of Osteopathic Medicine, Hattiesburg, USA

**Keywords:** complex regional pain syndrome, crps, omm, omt, osteopathic manipulative treatment

## Abstract

Complex regional pain syndrome (CRPS) is a chronic pain condition characterized by sensory, autonomic, and motor disturbances that can lead to significant functional impairment and decreased quality of life. The pathophysiology of CRPS is multifactorial and involves central and peripheral sensitization, autonomic dysfunction, and neurogenic inflammation. Current management for CRPS consists of pharmacologic medications for pain as well as injections and physical therapy. Physical therapy is a type of manual medicine that serves as a means of neuromodulation for pain to help patients suffering from CRPS maintain functionality and quality of life. Osteopathic manipulative therapy is another type of manual medicine whose theorized mechanisms seem to make it eligible for potential treatment of CRPS. The objective of this article is to evaluate and synthesize the available evidence regarding the use of osteopathic manipulative treatment for CRPS. A literature search was conducted utilizing MEDLINE/PubMed, Semantic Scholar, Google Scholar, Cochrane Library, and clinicaltrials.gov research databases. Articles were included if they involved patients with CRPS being treated with osteopathic manipulative treatments. A total of 404 articles were initially identified, of which four met the inclusion criteria. A risk of bias assessment was performed for the included studies. This scoping review found that evidence supporting the use of osteopathic manipulative treatment (OMT) for CRPS remains limited and heterogeneous. Further research utilizing larger, higher powered studies with standardized protocols and outcomes is required to define the potential role of OMT for patients with CRPS.

## Introduction and background

Complex regional pain syndrome (CRPS) is a chronic pain condition characterized by persistent pain and inflammation that typically develops following an injury or other traumatic events, such as surgery [1]. The condition most commonly affects the extremities and is often associated with pain that is disproportionate to the initial inciting event. Following tissue injury, inflammatory mediators are released, which sensitize peripheral pain receptors and amplify pain signaling [2,3]. As a result, persistent nociceptive input leads to neurogenic inflammation, which contributes to its symptoms. In addition to persistent pain, CRPS is frequently accompanied by sensory disturbances, vasomotor changes, edema, and motor dysfunction, all of which can significantly impair a patient’s functional ability and quality of life [1]. Several subtypes of CRPS exist and are generally categorized based on factors such as the presence or absence of nerve injury, clinical presentation, and disease progression [1]. Diagnosis of CRPS is primarily made using the Budapest Criteria, which incorporate both patient-reported symptoms and clinical examination findings [4]. These criteria evaluate multiple domains, including sensory abnormalities, vasomotor changes, edema or sudomotor dysfunction, and motor or trophic changes, in order to establish a diagnosis [4].

Current treatment guidelines for CRPS emphasize a multidisciplinary approach that includes both pharmacologic and non-pharmacologic interventions [5,6]. Pharmacologic management commonly includes medications such as nonsteroidal anti-inflammatory drugs, corticosteroids, gabapentin, bisphosphonates, and botulinum toxin A, which are used to target inflammation, neuropathic pain, and other aspects of the disease process [5]. In addition to pharmacologic treatments, several invasive non-pharmacologic interventions may be considered in refractory cases. These interventions include sympathetic nerve blocks, spinal cord stimulators, dorsal root ganglion stimulators, and, in rare cases, amputation for severe and debilitating disease. While these interventions may provide symptom relief for some patients, they are generally reserved for cases in which more conservative therapies have failed. Consequently, less invasive non-pharmacologic approaches are often considered earlier in the treatment course.

Currently, physical therapy is considered one of the first-line treatments for CRPS [5,7]. Physical therapy interventions aim to restore function, improve range of motion, and reduce pain through graded exercise, desensitization techniques, and manual therapies. The proposed pathophysiologic mechanism for the use of physical therapy involves neuromodulation through the reduction of inappropriate proprioceptive hyperactivity as well as the activation or inhibition of descending pain pathways [8]. Additionally, it has been proposed that physical therapy may produce analgesic effects through the release of neuropeptides such as neurotensin and oxytocin, which may provide short-term reductions in pain perception [5,7]. Through these mechanisms, physical therapy and rehabilitative interventions can help preserve functional ability and maintain quality of life in individuals with CRPS [5]. Improvement in pain and mobility through these interventions may also allow patients to participate in additional therapies that they may not otherwise tolerate due to severe pain or functional limitation. As such, manual medicine currently plays an important role in the management of patients with CRPS.

Another form of manual medicine is osteopathic manipulative therapy. Osteopathic medicine focuses on the diagnosis and treatment of somatic dysfunction, which refers to impaired or altered function of related components of the body framework, including fascia, muscles, bones, ligaments, and visceral structures [9]. Myofascial release, a commonly used osteopathic manipulative technique, has been shown to influence fascial mobility and demonstrate measurable reductions in pain following treatment [10,11]. Myofascial release is proposed to work by improving fascial elasticity through the treatment of dense collagen within fascia that may develop following trauma, infection, or inflammation. By restoring elasticity and reducing fascial tension, this technique may influence nociceptive signaling and reduce pain [11,12]. Another treatment modality developed by Dr. Lawrence Jones, an osteopathic physician, is counterstrain. This technique functions based on the theory of decreasing proprioceptive hyperactivity, thereby allowing affected tissues to return to normal neuromuscular function [8].

CRPS is a multifactorial condition involving central sensitization, autonomic dysfunction, inflammatory changes, and altered microcirculation [1-3]. Osteopathic manipulative treatment (OMT) has been proposed to influence autonomic regulation, lymphatic and vascular flow, inflammatory processes, and pain modulation, suggesting a potential therapeutic role in patients with CRPS in addition to its effects on the musculoskeletal and fascial systems [10,11]. Despite these proposed mechanisms, there has been no comprehensive review evaluating the role of OMT in CRPS, and the current state of evidence and proposed clinical applications remain unclear. Therefore, this scoping review aims to evaluate and synthesize the available literature on the use of OMT in the management of CRPS.

## Review

Materials and methods

Search Strategy

Studies were obtained from the MEDLINE/PubMed, Semantic Scholar, Google Scholar, Cochrane Library, and ClinicalTrials.gov research databases. This scoping review was conducted and reported in accordance with the Preferred Reporting Items for Systematic Reviews and Meta-Analyses Extension for Scoping Reviews (PRISMA-ScR) guidelines [13]. Searches were conducted from database inception through March 8, 2026, using combinations of terms related to complex regional pain syndrome and osteopathic manipulative treatment, including “CRPS,” “complex regional pain syndrome,” “osteopathic manipulative treatment,” “osteopathic manipulation,” “manual therapy,” “counterstrain,” and “myofascial release.” Search strategies were optimized for each database to account for differences in database indexing and search functions while maintaining a consistent focus on CRPS and OMT. Google Scholar and Semantic Scholar searches returned a large number of results; therefore, results were screened by relevance, and the first 200 results were reviewed for eligibility, as these databases rank results by relevance.

Eligibility Criteria

Studies were found to be eligible for inclusion if they involved patients with CRPS and utilized OMT as an intervention. All articles other than reviews that included OMT performed on CRPS patients were considered eligible for inclusion, regardless of study design, clinical context, or outcomes measured. Studies evaluating non-osteopathic manual therapies were excluded.

Study Selection and Data Extraction

**Figure 1:**
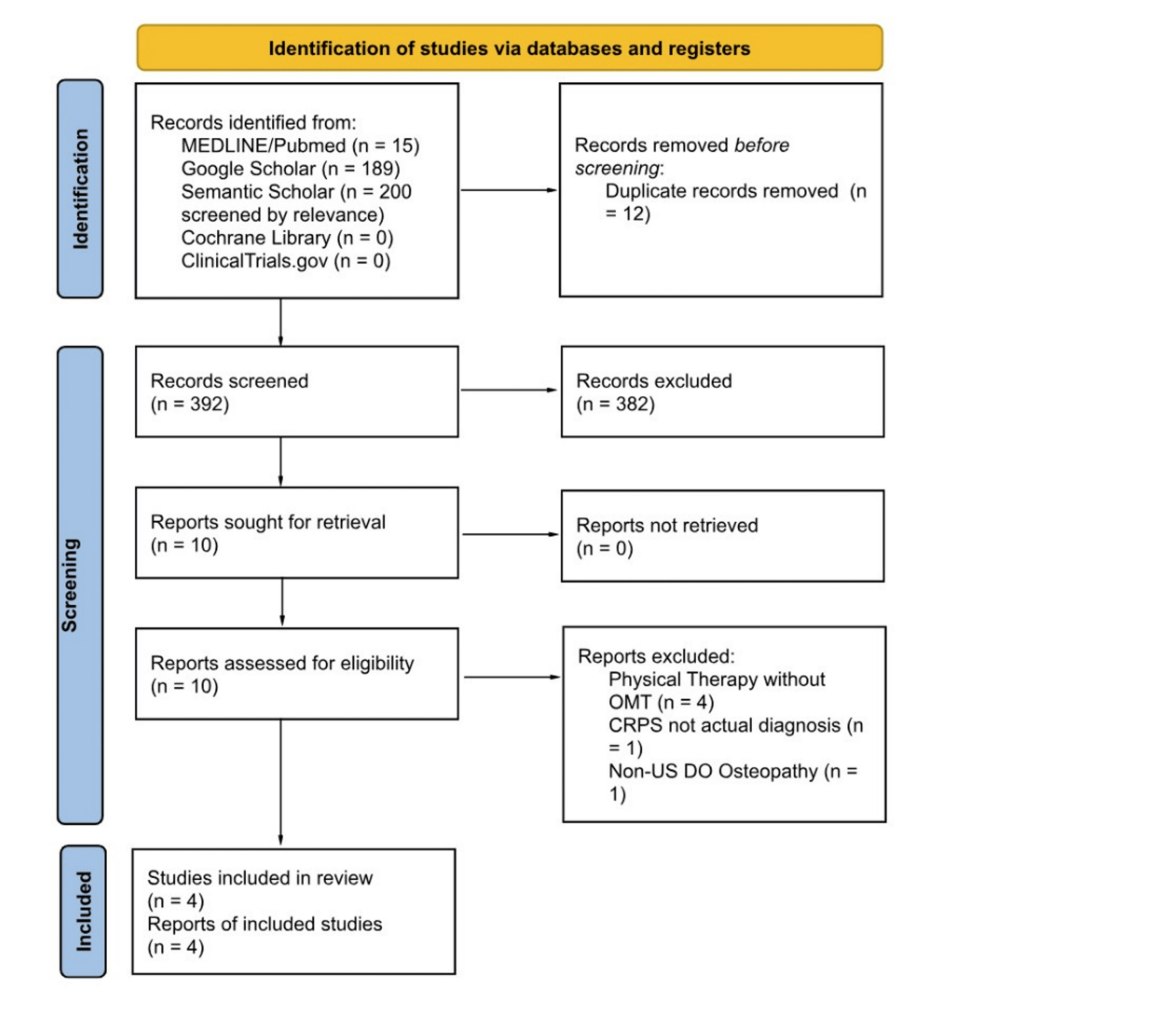
PRISMA Flow Diagram for the Selection of Studies PRISMA: Preferred Reporting Items for Systematic Reviews and Meta-Analyses; OMT: osteopathic manipulative treatment; CRPS: complex regional pain syndrome

Two authors (A.P. and P.N.) performed independent literature searches and study selection on MEDLINE/PubMed, Google Scholar, Semantic Scholar, the Cochrane Library, and clinicaltrials.gov. Data extraction was performed using a standardized data collection form. Any disagreements between reviewers were resolved by consensus. Risk-of-bias assessments were applied at the study level using predefined criteria. Titles and abstracts were screened for relevance, followed by a full-text review of potentially eligible studies. Reasons for exclusion after full text retrieval are detailed in the PRISMA 2020 flow diagram (Figure 1) [13]. For included articles, information regarding author, year of publication, type of article, sample size, outcomes measured, and reported outcomes was extracted. Due to significant heterogeneity of design and outcomes, results were synthesized narratively. Study selection is summarized in Figure 1. This scoping review was conducted and reported utilizing PRISMA-ScR as a basis for evidence gathering [13].

Results

A total of 404 records were identified through the initial literature search. After screening, 10 articles were retrieved and assessed for eligibility. All 10 underwent full-text review, and four were included in the scoping review. While many articles mentioned CRPS, few evaluated OMT as a treatment modality. Several articles identified during the search mentioned CRPS as part of the differential diagnosis but did not include patients with a confirmed diagnosis of CRPS and were therefore excluded. Studies where OMT adjacent techniques were used by non-osteopathic physicians were excluded due to differences between OMT and the aforementioned similar techniques. Summaries of the included articles, including type of paper, sample size, outcomes measured, and reported outcomes, are presented in Table 1. Critical appraisal of the included studies, including summaries of the study design, the appraisal tools used, and the key limitations identified, is presented in Table 2.

**Table 1:** Summary of Articles Included in the Scoping Review Evaluating Osteopathic Manipulative Treatment (OMT) for CRPS Information presented includes study design, sample size, outcomes measured, and reported outcomes. OMT: osteopathic manipulative treatment; CRPS: complex regional pain syndrome; ROM: range of motion; TMJ: temporomandibular joint; FDM: fascial distortion model.

Author, Year	Type of Paper	Sample Size (n)	Outcomes Measured	Reported Outcomes
Fischer et al., 2009 [14]	Controlled observational study	40 (20 CRPS, 20 controls)	Hip range of motion (ROM), temporomandibular index, and pain	TMJ myofascial release significantly altered hip ROM, particularly in CRPS patients, suggesting a functional relationship between jaw dysfunction and hip mobility.
Schranz et al., 2020 [15]	Case report	1	Pain, ROM, neuropathy symptoms, and proprioception	A 23-year-old woman with CRPS following foot surgery was treated with a novel counterstrain-based OMT approach targeting neuropathic symptoms. After six sessions, she experienced significant symptom resolution and improved proprioception and temperature discrimination.
Kincheloe et al., 2021 [16]	Case report	1	Functional mobility, pain, ROM, and wheelchair-to-bed transfer	A 94-year-old nursing home patient with CRPS and severe mobility limitations was treated with the fascial distortion model. Shoulder pain resolved, hip extension improved, and transfer time improved from ~15s with assistance to ~10s with minimal assistance with sustained improvement for ~two months.
Deol et al., 2021 [17]	Case report	1	Pain score, mobility, daily activity function, and psychological well-being	A 52-year-old woman with CRPS after ankle trauma received integrative osteopathic care including OMT and supportive therapies. Over ~1.5 years, pain decreased from ~7–8/10 to ~1/10 and the patient regained mobility and returned to full-time work.

**Table 2:** Risk of Bias Assessment and Methodological Limitations of Articles Included in the Scoping Review The Joanna Briggs Institute critical appraisal tools were used to assess methodological quality of included studies [18]. OMT: osteopathic manipulative treatment; CRPS: complex regional pain syndrome; JBI: Joanna Briggs Institute.

Author (Year)	Study Design	Appraisal Tool Used	Key Limitations Identified
Fischer et al., 2009 [14]	Controlled observational study	JBI Critical Appraisal Checklist for Quasi-Experimental Studies	Small sample size; limited control of confounders; short-term outcome assessment; lack of blinding; unclear external validity.
Schranz et al., 2020 [15]	Case report	JBI Critical Appraisal Checklist for Case Reports	Single patient; no control group; subjective outcome reporting; limited follow-up; results not generalizable.
Kincheloe et al., 2021 [16]	Case report	JBI Critical Appraisal Checklist for Case Reports	Single elderly patient; subjective functional measures; lack of standardized mobility metrics; potential practitioner bias; limited reproducibility.
Deol et al., 2021 [17]	Case report	JBI Critical Appraisal Checklist for Case Reports	Multimodal treatment approach prevents isolation of OMT effects; long treatment period with multiple interventions; subjective patient-reported outcomes.

Discussion

This scoping review identified a small number of published reports evaluating the use of OMT in patients with CRPS. The available literature consists primarily of case reports and small observational studies, with significant variability in treatment techniques, outcome measures, and patient populations. Despite this heterogeneity, several common treatment approaches were identified across the included studies, including fascial-based therapies, indirect techniques such as counterstrain, and multimodal treatment approaches incorporating OMT as an adjunctive therapy.

Fascial-Based Therapies

Fascial-based techniques were among the most commonly described osteopathic interventions across the included studies, particularly myofascial release and fascial distortion techniques [15,17]. Across these reports, fascial-based therapies were associated with improvements in range of motion, mobility, and pain. These findings suggest that fascial restrictions may contribute to pain and functional limitation in CRPS, potentially through mechanisms such as impaired circulation, altered biomechanics, and nerve compression within fascial planes. Techniques aimed at improving fascial mobility and tissue glide may therefore help reduce mechanical irritation of peripheral nerves, improve local circulation, and decrease inflammatory signaling. Although the available evidence is limited, the consistency of reported improvements in mobility and pain across these cases suggests that fascial-based OMT may be a useful adjunctive approach in the management of CRPS-related musculoskeletal dysfunction [15,17].

Counterstrain

Indirect osteopathic techniques, particularly counterstrain, were described in cases where pain and hypersensitivity limited the use of more direct manual therapies [16]. In this case report, the patient experienced decreased pain and improved sensory function following treatment. Counterstrain is thought to reduce abnormal neuromuscular reflex activity and decrease nociceptive input by placing affected tissues in positions of comfort, which may help reduce peripheral and central sensitization. Given that CRPS is associated with central sensitization and abnormal pain processing, indirect techniques such as counterstrain may be particularly useful in patients with significant allodynia or hyperalgesia who may not tolerate more direct techniques [16].

Multi-technique Approaches

One included study described a multimodal treatment approach in which multiple OMT techniques were used in combination with other interventions, including nutritional support and lifestyle modifications [18]. In this case, the patient experienced improvements in mobility, pain, and quality of life. While this represents only a single report, it suggests that OMT may be used as part of a broader multidisciplinary treatment approach rather than as a standalone therapy. This is consistent with current CRPS management strategies, which emphasize multimodal care including physical therapy, pharmacologic management, and psychological support [18].

Overall, across the included studies, OMT was most commonly used to address pain, range of motion limitations, and functional impairment in patients with CRPS [15-18]. Techniques were often selected based on patient tolerance, with more indirect techniques used in patients with significant pain and hypersensitivity, and techniques aimed at improving mobility and tissue restriction used in patients with functional limitations. Despite variation in treatment techniques and outcome measures, most reports described improvements in pain and mobility following treatment. However, the heterogeneity in treatment approaches, outcome measures, and patient characteristics makes it difficult to determine which specific techniques are most effective or which patients may benefit most from treatment.

Limitations

The studies included in this paper have several limitations. Because the majority of the included articles are case reports, the findings cannot be generalized. Although several studies used pain as a measure to determine efficacy, few utilized a standardized scale or grading system to quantify pain levels. This heterogeneity further limits the ability to synthesize the results. Additionally, because most of the included articles are case reports, there are no control groups available for comparison. The lack of control groups is also associated with small sample sizes, as the included case reports each feature a single individual, and Fischer et al. included only 20 patients treated with OMT [14]. Furthermore, the included articles did not use standardized treatment protocols or practitioner techniques for OMT, with the majority focusing on somatic dysfunction. Additionally, the CRPS stage was not consistently reported across the included studies, making it difficult to determine whether disease stage influenced treatment selection, tolerability of osteopathic manipulative treatment, or clinical outcomes. This is particularly relevant given that patients in earlier stages of CRPS may have significant allodynia and hyperalgesia that may limit tolerance to certain manual techniques, whereas patients in later stages may present with contractures and decreased mobility that could potentially benefit from manual interventions. Additionally, only one of the included studies specified whether patients had CRPS Type I or Type II, which further limits the ability to determine whether osteopathic manipulative treatment may be more appropriate for specific CRPS subtypes. Additionally, the search strategy occasionally identified articles in which CRPS was mentioned but was not the primary condition being treated, which reflects the broad nature of database search algorithms and the overlap in terminology used to describe neuropathic pain conditions. This may have contributed to the limited number of eligible studies identified after screening.

Several methodological limitations of this review should also be acknowledged. Although study selection screening was performed independently by two reviewers, the small number of available studies limits the strength of the conclusions. Additionally, the included studies varied substantially in study design, patient populations, outcome measures, and treatment protocols. This notable heterogeneity prevents quantitative synthesis and limits the analysis to a qualitative discussion of the findings. As a scoping review, this study was designed to map the existing literature rather than evaluate treatment efficacy, and therefore, no formal meta-analysis or pooled outcome analysis was performed. Finally, publication bias may also be present, as studies with negative findings are less likely to be published.

## Conclusions

Available literature regarding OMT for the management of CRPS remains limited. This review identified several articles that include a small number of heterogeneous publications, which consisted of studies with limited methodological rigor and case reports. As a result, it is not possible to make definitive conclusions regarding the effectiveness of OMT for CRPS. These findings illustrate a gap in the literature rather than providing strong guidance for clinical practice. Further research with larger sample sizes, standardized outcome measures, and further controlled study designs would be necessary to elucidate the potential role of OMT use in patients suffering from CRPS. Such investigations may help clarify the ability, or lack of, osteopathic interventions to have meaningful benefits for this patient population.

## Appendices

The MEDLINE/PubMed search combined text words and Medical Subject Headings (MeSH) as follows:

("Complex Regional Pain Syndrome"[Mesh] OR "complex regional pain syndrome"[tiab] OR CRPS[tiab]) AND ("osteopathic manipulation"[Mesh] OR "osteopathic manipulation"[tiab] OR "osteopathic manipulative treatment"[tiab] OR "osteopathic manual"[tiab] OR counterstrain[tiab] OR "strain counterstrain"[tiab] OR "myofascial release"[tiab] OR "manual therapy"[tiab])

The Google Scholar search used the query: "complex regional pain syndrome" "osteopathic manipulative treatment"

The Semantic Scholar search used: "complex regional pain syndrome" "osteopathic manipulative treatment"

The Cochrane Library search used: ("complex regional pain syndrome" OR CRPS OR "reflex sympathetic dystrophy")

AND

(osteopathic OR counterstrain OR "strain counterstrain" OR "myofascial release")

The search of ClinicalTrials.gov used “Complex Regional Pain Syndromes” as the condition/disease and “osteopathic OR "osteopathic manipulative treatment" OR Counterstrain” in the other terms selection.
